# Microwave-assisted green synthesis of anilines, phenols, and benzenediamines without transition metals, ligands, or organic solvents

**DOI:** 10.1080/17518253.2018.1486464

**Published:** 2018-07-03

**Authors:** N. McConnell, B. Frett, H. Li

**Affiliations:** a Department of Pharmacology and Toxicology, University of Arizona, Tucson, AZ, USA; b Department of Pharmaceutical Sciences, University of Arkansas for Medical Sciences, Little Rock, AR, USA

**Keywords:** Amination, hydroxylation, reduction, benzenediamine, aminophenol

## Abstract

A novel, microwave-assisted method producing anilines and phenols from activated aryl halides is reported. This high-yielding method reduces current reaction requirements and removes organic solvents and catalysts making a more efficient and eco-friendly alternative for the synthesis of important pharmaceutical building blocks.

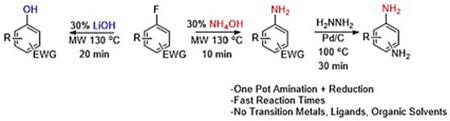

## Introduction

Aromatic amines and phenols are found abundantly in medicinally relevant compounds. The ease at which they form bonds has made them heavily utilized for drug synthesis and, due to their high value, efficient methods for their production have been long sought after. Traditionally, amination and hydroxylation reactions involved the use of liquid ammonia or concentrated strong bases, high pressure, high temperature, and long reaction times (*1*) and, in the case of phenols, sometimes in two steps (*2*).

There have been significant efforts to improve the safety and efficiency of traditional methods for aminations and hydroxylations. One common method was employing *metal* catalysts, typically palladium (*3–10*), copper (*11–19*), nickel (*20*), or iron (21). While these made improvements by decreasing temperature and pressure, the reactions still require organic solvents and lengthy reaction times. Although *meta*l-catalysis permits the facile synthesis of anilines and phenols, environmental impact warrants identification of greener conditions.

Work has been completed to decrease the environ-mental burden of aminations and hydroxylations. Multiple groups have reported methods using green ligands (*22–24*) or green solvents like ammonia (*24*) or water (*13,21, 25–27*). These works represent a significant step in making amination and hydroxylation more ecofriendly but still requires the use of environmentally toxic copper. Further, many reaction durations are on the scale of 12 or more hours, significantly increasing iteration time for the synthesis of chemical libraries used for drug discovery programs.

The rise of microwave irradiation has allowed for many reactions to be revisited and further optimized due to its ability to steadily control high temperatures and pressures (*28*). However, it has rarely been utilized to aminate and hydroxylate activated aryl halides. Recently, Yu et al. (*29*) reported a microwave-assisted method for the hydroxylation of aryl halides, but similar to previous methods, still required *meta*l catalyst, ligand, and organic solvents.

**Scheme 1.**
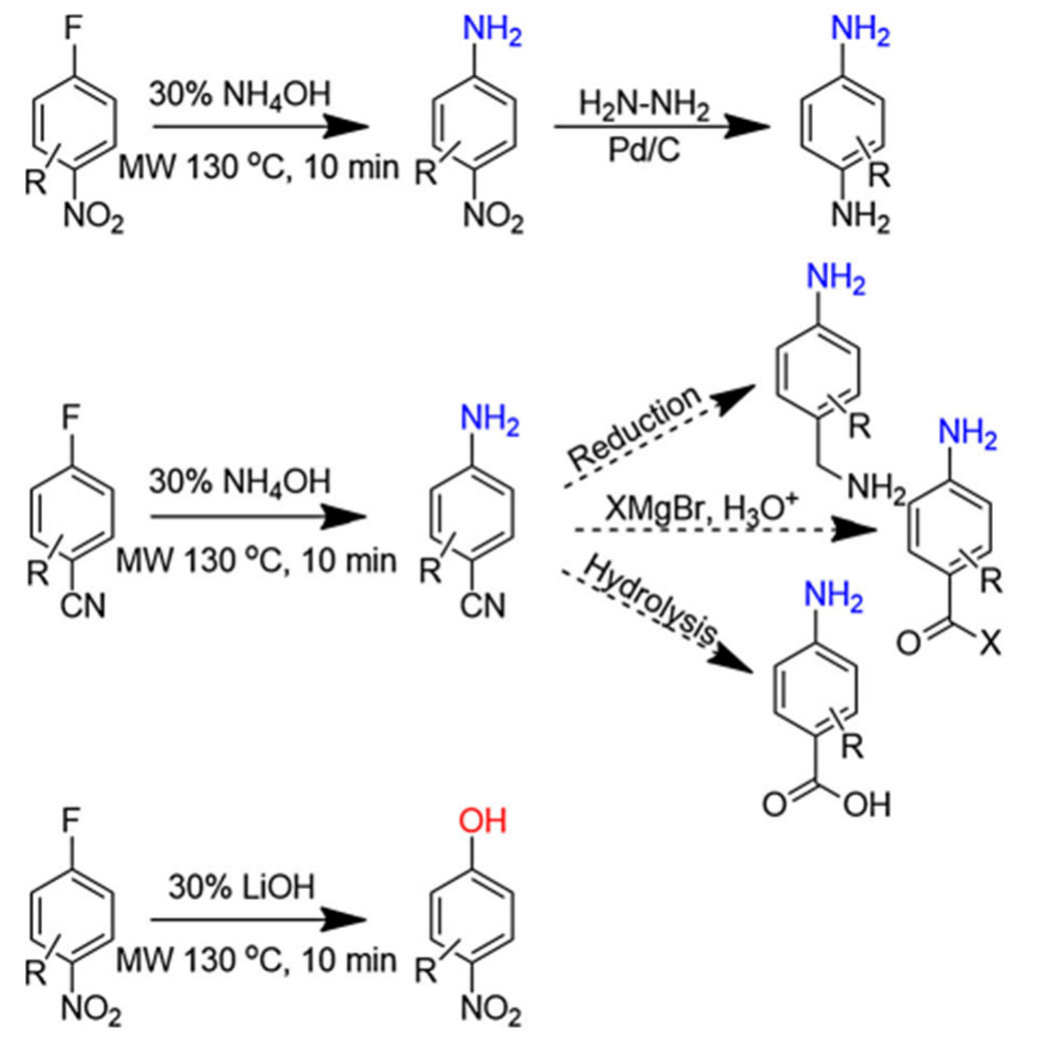
Synthetic routes to pharmaceutically relevant intermediates using the reported method.

Herein, we report a short duration, high yielding, nucleophilic aromatic substitution reaction that removes the need for catalysts and organic solvents (Scheme 1). We have achieved this through activated aryl halides in an aqueous ammonium hydroxide or lithium hydroxide solution under microwave irradiation. Additionally, we have developed a one-pot amination and reduction protocol to rapidly access benzenediamine analogs. This method increases the scope and efficiency of accessing important pharmaceutical building blocks.

This aromatic substitution technique improves on current protocols and has the potential for industrial scale-up. Briefly, it requires decreased reaction times with minimal purification efforts.To lessen environmental impact, organic solvents and *metal* catalysts have been substituted with simple aqueous solutions. From an industrial standpoint, the use of aqueous solutions is much safer, which avoids toxic, volatile, and/or combustible organic solvents. Also, reaction conversions are typically 100% making purification requirements nominal to help reduce cost and increase industrial adaptation.

## Results and discussion

Optimization studies for amination were carried out using 100 mg of **1a** in 2 mL of 28–30% ammonium hydroxide solution to determine the temperature that yielded complete conversion with the shortest reaction duration (Table 1). It was observed that heating at 110°C for 10 min was sufficient for complete conversion (Table 1, **entry 3**), but at 130°C, the reaction duration was decreased to as little as 5 min (Table 1, **entry 5**).

Following optimization, we explored the scope of the reaction. We altered the position of the fluorine group to confirm its reactivity to the *para* position (Table 2, **entry 2**) and its lack of reactivity to the *meta* position (Table 2, **entry 3**). Additional halogen substituents were added to test for any steric interference (Table 2, **entries 4–6**), and full conversion was still observed. The selectivity of fluorine in the reaction over other halogens was tested by placing bromine *para* and fluorine *ortho* to the nitro group (Table 2, **entry 7**). The fluorine underwent full conversion and no additional side products were observed. Furthermore, 100% conversion was still observed with the addition of electron donating methyl (Table 2, **entry 17**) and methoxy (Table 2, **entry 18**) groups *ortho* to fluorine.

With these conditions, it appeared the nitro group is necessary for full conversion. Replacing the nitro with a nitrile group furnished 80% conversion after heating to 140°C for 20 min with no detection of the hydrolysis product (Table 2, **entry 12**). Capacity for derivatization off the nitrile group greatly increases the utility of the aniline product for larger synthetic efforts. The addition of trifluoromethyl groups worked in the presence of nitro groups (Table 2, **entries 9 and 10**), but no conversion was observed if –CF_3_ was the sole electron withdrawing group (Table 2, **entries 13 and 14**).

Fluorine was successfully replaced by chlorine in one of the more activated samples (Table 2, **entry 15**). To achieve complete conversion, an increase in temperature to 140°C and additional heating time was required. The less activated chlorine example showed only 20% conversion under similar conditions (Table 2, **entry 16**). Attempting to go any higher in temperature led to reaction pressures that would be difficult to implement on an industrial scale.

To further examine the scope of the reaction, the reaction was performed in the presence of bulky aryl and heterocycle substituents on the nitro-fluoro-benzene starting material (Table 3). Full conversion was observed with starting materials containing the bulky substituents in the *meta* and *para* positions relative to the fluorine group were tolerated, although over half required additional heating times, up to 20 min (**entries 1, 5–7, 9, 11, 12**, Table 3). Interestingly, we did not observe any conversion when the additions were made *ortho* to the fluorine, even with additional time and increased heating. We believe this is simply an issue of steric hindrance.

To produce phenols, a similar procedure was developed using LiOH as the hydroxide source. Ten minutes of heating at 130°C showed incomplete conversion, and an additional 10 min of heating led to complete conversion. A small sample of compounds previously used were successfully converted into phenols (Table 4). Attempts of hydroxylation with weaker activating groups were still unsuccessful even when heating up to 160°C. This is likely a result of the decreased nucleophilicity of oxygen when compared with amine.

Finally, an efficient one-pot protocol was designed for amination and subsequent reduction to yield benzenediamines (Table 5). Following amination, reactions were heated to remove excess ammonia until the pH of the reaction mixture was neutral. A catalytic amount of 10% Pd/C was added and the reaction was sealed. Using a syringe and needle, hydrazine hydrate was added. Heating at 100°C for 20 min yielded complete conversion. Hydrazine hydrate is an effective, liquid hydrogen source that eliminates handling a flammable gas, allows for precise use of resources, and produces nitrogen gas as the sole byproduct. Six benzenediamine analogs were produced using the developed protocol.

## Conclusions

In conclusion, we have presented a novel and green method to produce substituted anilines, phenols, and benzenediamines. This method is a further testament to the value of microwave assistance in reactions to develop greener synthetic routes. Because of its simplicity in work-up, brevity in the reaction setting, and eco-friendliness, this new method may serve as a standard for industrial scale synthesis of anilines and phenols. The methodology will also be especially important in the medicinal chemistry and drug discovery field where the efficient transformation to amines and phenols is of great use for the formation of larger pharmaceutically active compounds.

## Experimental

### General

All solvents, reagents, and catalysts were commercially purchased and used without further purification. For products purified using flash chromatography, silica gel (0.035–0.070 mm, 60 Å) was used as the stationary phase, eluting with hexane/ethyl acetate mixtures. All microwave reactions were completed in microwave vials and used a Biotage Initiatior microwave synthesizer. ^1^HNMR spectra and ^13^CNMR spectra were recorded at 400 and 100 MHz, respectively, using a Varian 400 MHz instrument (Model# 4001S41ASP) for all reaction products.

### General procedure for the amination of aryl halides

100 mg (0.361–0.826 mmol) of starting material was added to 2 mL of 28–30% ammonium hydroxide solution in a microwave vial. The vial was sealed and heated in microwave at 130–140°C for 5–20 min, until thin layer chromatography (TLC) showed complete conversion. The product was extracted in a *separatory* funnel using dichloromethane or dichloromethane/isopropanol 4:1. The organic layer was dried using Na_2_SO_4_ and solvent removed under reduced pressure.

### General procedure for the hydroxylation of aryl halides

100 mg (0.458–0.826 mmol) of starting material was added to 2 mL of 30% lithium hydroxide solution in a microwave vial. The vial was sealed and heated in microwave at 130–140°C for 5–20 min, until TLC showed complete conversion. The product was extracted in a se*para*tory funnel using dichloromethane or dichloromethane/isopropanol 4:1. The organic layer was dried using Na_2_SO_4_ and solvent removed under reduced pressure.

### General procedure for the synthesis of starting materials 17a-28a

A solution of the aryl halide (250 mg, 1.136 mmol), the boronic acid (1.136 mmol), Pd_2_(dba)_3_ (20.81 mg, 0.023 mmol), P(Cy)_3_ (19.12 mg, 0.068 mmol), and Na_2_CO_3_ (482 mg, 4.55 mmol) in argon flushed DMF/H_2_O 4:1 (5 mL) was heated at 85°C for 16 h. The reaction mixture was cooled to room temperature and diluted with ethyl acetate. The organic layer was washed with saturated NaHCO_3_ (3 × 15 mL) and deionized water (3×15 mL). The organic layer was dried over Na_2_SO_4_ and solvent removed under reduced pressure. The crude product was purified using flash chromatography (SiO_2_, hexane/ethyl acetate).

### General procedure for the synthesis of benzenediamines

100 or 200 mg (0.584–0.956 mmol) of starting material was added to 2 mL of 28–30% ammonium hydroxide solution in a microwave vial. The vial was sealed and heated in microwave at 130–140°C for 5–20 min, until TLC showed complete conversion. Following, the reaction solution was refluxed with conventional heating unsealed for 20 min or until the reaction pH was neutral. A catalytic amount of 10% Pd/C was added and the reaction vessel was re-sealed. Hydrazine (10 EQ) was added and the reaction was heated with conventional heating to 100°C for 20 min, until TLC showed complete conversion. The reaction was filtered over celite to remove Pd/C and solvent removed under reduced pressure. The crude product was purified using flash chromatography (SiO_2_, hexane/ethyl acetate).

### Physical and spectroscopic data of isolated products

#### 2-nitroaniline (**1b**)

Orange solid, 93%. ^1^H NMR (400 MHz, Chloroform-*d*) δ 8.12 (dd, *J* = 8.6, 1.4 Hz, 1H), 7.40–7.31 (m, 1H), 6.81 (dd, *J* = 8.4, 1.1 Hz, 1H), 6.71 (ddd, *J* = 8.6, 7.0, 1.2 Hz, 1H), 6.4 (s, 2H). ^13^C NMR (100 MHz, Chloroform-*d*) δ 144.76, 135.69, 132.12, 126.11, 118.80, 116.90.

#### 4-nitroaniline (**2b**)

Yellow solid, 94% (15% when completed with 4-chloronitrobenzene). ^1^H NMR (400 MHz, Chloroform-*d*) δ 8.07 (d, *J* = 9.0 Hz, 2H), 6.63 (d, *J* = 9.0 Hz, 2H), 4.41 (s, 2H). ^13^C NMR (100 MHz, Chloroform-*d*) δ 152.49, 126.34, 115.69, 113.36.

#### 2-fluoro-4-nitroaniline (**4b**)

Yellow solid, 98%%. ^1^H NMR (400 MHz, Chloroform-*d*) δ 7.93 (m, 2H), 6.77 (m, 1H), 4.45 (s, 1H). ^13^C NMR (100 MHz, Chloroform-*d*) δ 149.04 (d, *J* = 242.8 Hz), 141.50, 141.38, 121.70 (d, *J* = 2.7 Hz), 114.26 (d, *J* = 4.2 Hz), 111.84 (d, *J* = 23.0 Hz).

#### 2-chloro-4-nitroaniline (**5b**)

Yellow solid, 99%. ^1^H NMR (400 MHz, Chloroform-*d*) δ 8.21 (d, *J* = 2.5 Hz, 1H), 8.00 (dd, *J* = 9.0, 2.5 Hz, 1H), 6.76 (d, *J* = 9.0 Hz, 1H), 4.82 (s, 2H). ^13^C NMR (100 MHz, Chloroform-*d*) δ 148.82, 125.96, 124.33, 117.70, 113.68.

#### 2-bromo-4-nitroaniline (**6b**)

Yellow solid, 86%. ^1^H NMR (400 MHz, Chloroform-*d*) δ 8.37 (d, *J* = 2.5 Hz, 1H), 8.03 (dd, *J* = 9.0, 2.5 Hz, 1H), 6.75 (d, *J* = 9.0 Hz, 1H), 4.86 (s, 2H). ^13^C NMR (100 MHz, Chloroform-*d*) δ 149.93, 138.90, 129.17, 124.89, 113.46, 106.94.

#### 5-bromo-2-nitroaniline (**7b**)

Orange solid, 97%. ^1^H NMR (400 MHz, Chloroform-*d*) δ 7.98 (d, *J* = 9.1 Hz, 1H), 7.01 (d, *J* = 2.0 Hz, 1H), 6.82 (dd, *J* = 9.1, 2.0 Hz, 1H), 6.10 (s, 2H). ^13^C NMR (100 MHz, Chloroform-*d*) δ 145.08, 130.61, 127.55, 120.92, 120.38, 120.10.

#### 4-bromo-2-nitroaniline (**8b**)

Orange solid, 96%δ. ^1^H NMR (400 MHz, Chloroform-*d*) δ 8.28 (d, *J* = 2.3 Hz, 1H), 7.43 (dd, *J* = 8.9, 2.3 Hz, 1H), 6.73 (d, *J* = 8.9 Hz, 1H), 6.08 (s, 2H). ^13^C NMR (100 MHz, Chloroform-*d*) δ 143.59, 138.46, 132.42, 128.25, 120.34, 107.80.

#### 4-nitro-2-(trifluoromethyl)aniline (**9b**)

Yellow solid, 84%%. ^1^H NMR (400 MHz, Chloroform-*d*) δ 8.40 (d, *J* = 2.5 Hz, 1H), 8.19 (dd, *J* = 9.0, 2.5 Hz, 1H), 6.77 (d, *J* = 9.0 Hz, 1H), 4.92 (s, 2H). ^13^C NMR (100 MHz, Chloroform-*d*) δ 149.86, 137.86, 128.66, 124.13 (q, *J* = 5.5 Hz), 123.59 (q, *J* = 272.5 Hz) 116.39, 112.26 (q, *J* = 31.9 Hz).

#### 2-nitro-4-(trifluoromethyl)aniline (**10b**)

Yellow solid, 99%%. ^1^H NMR (400 MHz, Chloroform-*d*) δ 8.43 (d, *J* = 2.1 Hz, 1H), 7.56 (dd, *J* = 8.8, 2.1 Hz, 1H), 6.92 (d, *J* = 8.8 Hz, 1H), 6.38 (s, 2H). ^13^C NMR (100 MHz, Chloroform-*d*) δ 146.46, 137.08, 131.64 (q, *J* = 3.2 Hz), 124.39 (q, *J* = 4.4 Hz) 123.40 (q, *J* = 270.9 Hz), 119.43.

#### 2,4-dinitroaniline (**11b**)

Pale Yellow solid, 96%%. ^1^H NMR (400 MHz, DMSO-*d6*) δ 8.79 (d, *J* = 2.7 Hz, 1H), 8.39 (s, 2H), 8.17 (dd, *J* = 9.4, 2.7 Hz, 1H), 7.12 (d, *J* = 9.4 Hz, 1H). ^13^C NMR (100 MHz, DMSO-*d6*) δ 150.24, 135.52, 129.72, 129.08, 123.81, 120.20.

#### 4-amino-2-bromobenzonitrile **(12b)**

White solid, 78%. ^1^H NMR (400 MHz, Chloroform-*d*) δ 7.38 (d, *J* = 8.5 Hz, 1H), 6.89 (d, *J* = 2.3 Hz, 1H), 6.58 (dd, *J* = 8.5, 2.3 Hz, 1H), 4.24 (s, 2H). ^13^C NMR (100 MHz, Chloroform-*d*) δ 151.80, 135.84, 132.25, 132.16, 127.03, 118.43, 113.77.

#### 2-nitro-4-(trifluoromethyl)aniline **(15b)**

Yellow solid, 90%%. ^1^H NMR (400 MHz, Chloroform-*d*) δ 8.43 (s, 1H), 7.56 (dd, *J* = 8.8, 2.0 Hz, 1H), 6.90 (d, *J* = 8.8 Hz, 1H), 6.35 (s, 2H). ^13^C NMR (100 MHz, Chloroform-*d*) δ 146.46, 137.08, 131.64 (q, *J* = 3.2 Hz), 124.39 (q, *J* = 4.4 Hz) 123.40 (q, *J* = 270.9 Hz), 119.43.

#### 2-methyl-4-nitroaniline **(17b)**

Yellow solid, 99%. ^1^H NMR (400 MHz, CDCl_3_) δ 8.02–7.97 (m, 2H), 6.65 (d, *J* = 8.7 Hz, 1H), 4.34 (s, 2H), 2.24 (s, 3H).

#### 2-methoxy-4-nitroaniline **(18b)**

Yellow solid, 84%. ^1^H NMR (400 MHz, CDCl_3_) δ 7.84 (dd, *J* = 8.7, 2.4 Hz, 1H), 7.69 (d, *J* = 2.4 Hz, 1H), 6.66 (d, *J* = 8.7 Hz, 1H), 4.54 (s, 2H), 3.97 (s, 3H).

#### 3-(4-fluoro-3-nitrophenyl)thiophene **(19a)**

Brown/orange solid, 62.1%, 157.5 mg. ^1^H NMR (400 MHz, Chloroform-*d*) δ 8.26 (dd, *J* = 7.0, 2.3 Hz, 1H), 7.85–7.81 (m, 1H), 7.55–7.50 (m, 1H), 7.48-7.45 (m, 1H), 7.38–7.36 (m, 1H), 7.33 (dd, *J* = 10.5, 8.7 Hz, 1H). ^13^C NMR (100 MHz, Chloroform-*d*) δ 154.44 (d, *J* = 264.9 Hz), 138.74, 133.04 (d, *J* = 8.3 Hz), 128.96, 128.38, 127.41, 125.79, 123.47 (d, *J* = 2.9 Hz), 121.98 (d, *J* = 1.0 Hz), 118.81 (d, *J* = 21.1 Hz).

#### 2-nitro-4-(thiophen-3-yl)aniline **(19b)**

Red solid, 99%. ^1^H NMR (400 MHz, DMSO-*d6*) δ 8.23 (d, *J* = 2.2 Hz, 1H), 7.84–7.78 (m, 2H), 7.64–7.60 (m, 1H), 7.54–7.49 (m, 3H), 7.08 (d, *J* = 8.8 Hz, 1H). ^13^C NMR (100 MHz, DMSO-d6) δ 145.70, 140.11, 134.52, 130.61, 127.64, 126.09, 123.54, 121.99, 120.30, 120.11.

#### 2-(4-fluoro-3-nitrophenyl)thiophene **(20a)**

Brown solid, 50.4%, 127.9 mg.^1^H NMR (400 MHz, Chloroform-*d*) δ 8.26 (dd, *J* = 6.9, 2.4 Hz, 1H), 7.85-7.81 (m, 1H), 7.39–7.28 (m, 3H), 7.13 (dd, *J* = 5.1, 3.7 Hz, 1H). ^13^C NMR (100 MHz, Chloroform-*d*) δ 154.49 (d, *J* = 265.5 Hz), 140.32, 132.43 (d, *J* = 8.3 Hz), 131.73 (d, *J* = 4.4 Hz), 128.50, 126.54, 124.78, 122.81, 118.97 (d, *J* = 21.4 Hz).

#### 2-nitro-4-(thiophen-2-yl)aniline **(20b)**

Red solid, 99%%. ^1^H NMR (400 MHz, Chloroform-*d*) δ 8.36 (d, *J* = 2.2 Hz, 1H), 7.62 (dd, *J* = 8.7, 2.2 Hz, 1H), 7.26–7.23 (m, 2H), 7.07 (dd, *J* = 5.1, 3.7 Hz, 1H), 6.84 (d, *J* = 8.7 Hz, 1H), 6.13 (s, 2H). ^13^C NMR (100 MHz, Chloroform-*d*) δ 143.71, 142.26, 133.58, 128.08, 124.41, 124.15, 122.69, 122.64, 119.31.

#### 4’-fluoro-2,5-dimethoxy-3’-nitro-1,1‘-biphenyl **(21a)**

Yellow solid, 40.3%, 127.0 mg. ^1^H NMR (400 MHz, Chloroform-d) δ 8.22 (dd, *J* = 7.2, 2.3 Hz, 1H), 7.80-7.76 (m, 1H), 7.29 (dd, *J* = 10.7, 8.7 Hz, 1H), 6.96–6.83 (m, 3H). ^13^C NMR (100 MHz, Chloroform-*d*) δ 154.49 (d, *J* = 264.7 Hz), 153.85, 150.45, 136.50 (d, *J* = 8.4 Hz), 135.30 (d, *J* = 4.4 Hz), 127.72, 126.84 (d, *J* = 2.8 Hz), 117.81 (d, *J* = 20.8 Hz), 116.40, 114.28, 112.60, 56.13, 55.83.

#### 2’,5’-dimethoxy-3-nitro-[1,1‘-biphenyl]-4-amine **(21b)**

Orange solid, 99%%. ^1^H NMR (400 MHz, Chloroform-*d*) δ 8.31 (d, *J* = 1.8 Hz, 1H), 7.61 (dd, *J* = 8.6, 1.8 Hz, 1H), 6.92-6.82 (m, 4H), 6.10 (s, 2H), 3.81 (s, 3H), 3.77 (s, 3H). ^13^C NMR (100 MHz, Chloroform-*d*) δ 153.84, 150.71, 143.69, 137.22, 132.01, 129.17, 127.44, 126.32, 118.27, 116.09, 113.23, 112.57, 56.23, 55.82.

#### 4,4’-difluoro-3-nitro-1,1’-biphenyl **(22a)**

Yellow solid, 69.7%, 186.4 mg. ^1^H NMR (400 MHz, Chloroform-*d*) δ 8.18 (dd, *J* = 7.0, 2.4 Hz, 1H), 7.81–7.78 (m, 1H), 7.57–7.49 (m, 2H), 7.36 (dd, *J* = 10.5, 8.7 Hz, 1H), 7.20–7.12 (m, 2H). ^13^C NMR (100 MHz, Chloroform-*d*) δ 163.06 (d, *J* = 248.9 Hz), 154.81 (d, *J* = 265.4 Hz), 137.32 (d, *J* = 4.3 Hz), 133.84 (d, *J* = 3.3 Hz), 133.68 (d, *J* = 8.4 Hz), 128.74 (d, *J* = 8.2 Hz), 124.19 (d, *J* = 2.7 Hz), 118.88 (d, *J* = 21.1 Hz), 116.22 (d, *J* = 21.8 Hz).

#### 4’-fluoro-3-nitro-[1,1‘-biphenyl]-4-amine **(22b)**

Orange solid, 99%%. ^1^H NMR (400 MHz, Chloroform-*d*) δ 8.32 (d, *J* = 2.1 Hz, 1H), 7.59 (dd, *J* = 8.6, 2.1 Hz, 1H), 7.53–7.47 (m, 2H), 7.16–7.08 (m, 2H), 6.89 (d, *J* = 8.6 Hz, 1H), 6.10 (s, 2H). ^13^C NMR (100 MHz, Chloroform-*d*) δ 162.36 (d, *J* = 246.8 Hz), 143.70, 134.94, 134.31, 129.40, 127.91 (d, *J* = 8.1 Hz), 123.76, 119.36, 115.83 (d, *J* = 21.5 Hz).

#### 4-fluoro-4’-methoxy-3-nitro-1,1‘-biphenyl **(23a)**

Orange solid, 72.5%, 203.8 mg. ^1^H NMR (400 MHz, Chloroform-*d*) δ 8.21 (dd, *J* = 7.0, 2.4 Hz, 1H), 7.80–7.76 (m, 1H), 7.53–7.48 (m, 2H), 7.33 (dd, *J* = 10.5, 8.7 Hz, 1H), 7.03–6.98 (m, 2H), 3.87 (s, 3H). ^13^C NMR (100 MHz, Chloroform-*d*) δ 160.03, 154.40 (d, *J* = 264.3 Hz), 143.28, 133.29 (d, *J* = 8.3 Hz), 128.95, 128.38, 128.08, 123.66 (d, *J* = 2.9 Hz), 118.66 (d, *J* = 21.0 Hz), 114.61, 55.40.

#### 4’-methoxy-3-nitro-[1,1‘-biphenyl]-4-amine **(23b)**

Red solid, 99%%. ^1^H NMR (400 MHz, Chloroform-*d*) δ 8.31 (d, *J* = 2.2 Hz, 1H), 7.59 (dd, *J* = 8.6, 2.2 Hz, 1H), 7.47 (d, *J* = 8.7 Hz, 2H), 6.96 (d, *J* = 8.7 Hz, 2H), 6.87 (d, *J* = 8.6 Hz, 1H), 6.07 (s, 2H), 3.84 (s, 3H). ^13^C NMR (100 MHz, Chloroform-*d*) δ 134.25, 128.95, 128.38, 128.10, 127.38, 123.20, 119.24, 114.62, 114.36, 55.37.

#### 1-(4-fluoro-3-nitrophenyl)naphthalene **(24a)**

Pale yellow solid, 62.6%, 190.0 mg. ^1^H NMR (400 MHz, Chloroform-*d*) δ 8.11 (dd, *J* = 7.1, 1.9 Hz, 1H), 7.90–7.84 (m, 2H), 7.70 (d, *J* = 8.4 Hz, 1H), 7.68–7.63 (m, 1H), 7.52–7.41 (m, 3H), 7.36–7.30 (m, 2H). ^13^C NMR (100 MHz, Chloroform-*d*) δ 154.81 (d, *J* = 265.1 Hz), 137.75 (d, *J* = 4.4 Hz), 136.93 (d, *J* = 8.4 Hz), 136.39, 133.80, 131.05, 128.99, 128.64, 127.32, 127.21 (d, *J* = 2.7 Hz), 126.88, 126.28, 125.33, 124.81, 118.44, 118.24.

#### 4-(naphthalen-1-yl)-2-nitroaniline **(24b)**

Orange solid, 99%%. ^1^H NMR (400 MHz, Chloroform-*d*) δ 8.28 (s, 1H), 7.91 (d, *J* = 7.8 Hz, 1H), 7.86 (dd, *J* = 8.3, 4.2 Hz, 2H), 7.56–7.38 (m, 5H), 6.93 (d, *J* = 8.5 Hz, 1H), 6.16 (s, 2H). ^13^C NMR (100 MHz, Chloroform-*d*) δ 143.83, 137.78, 137.59, 133.87, 131.46, 129.74, 128.49, 128.04, 126.99, 126.93, 126.35, 125.95, 125.42, 125.36, 124.80, 118.67.

#### 3,4’-difluoro-4-nitro-1,1’-biphenyl **(25a)**

Brown/orange solid. 62.1%, 166 mg. ^1^H NMR (400 MHz, Chloroform-*d*) δ 8.16 (t, *J* = 8.1 Hz, 1H), 7.61–7.56 (m, 2H), 7.47 (s, 1H), 7.46–7.43 (m, 1H), 7.23–7.17 (m, 2H). ^13^C NMR (101 MHz, Chloroform-*d*) δ 163.63 (d, *J* = 250.4 Hz), 155.88 (d,*J* = 265.0 Hz), 148.12, 133.69, 129.10 (d, *J* = 8.5 Hz), 127.56, 126.75 (d, *J* = 2.4 Hz), 122.69 (d, *J* = 3.6 Hz), 116.44 (d, *J* = 21.7 Hz), 116.37 (d, *J* = 21.8 Hz).

#### 4’-fluoro-4-nitro-[1,1‘-biphenyl]-3-amine **(25b)**

Yellow solid, 99%.’ ^1^H NMR (400 MHz, Chloroform-d) δ 8.18 (d, *J* = 8.9 Hz, 1H), 7.57–7.52 (m, 2H), 7.18–7.12 (m, 2H), 6.93 (d, *J* = 1.9 Hz, 1H), 6.88 (dd, *J* = 8.9, 1.9 Hz, 1H), 6.15 (s, 2H). ^13^C NMR (100 MHz, DMSO-*d6*) δ 163.04 (d, *J* = 246.4 Hz), 146.82, 146.29, 135.19 (d, *J* = 3.1 Hz), 129.94, 129.40 (d, *J* = 8.4 Hz), 126.70, 116.82, 116.40 (d, *J* = 21.6 Hz), 114.81.

#### 1-(3-fluoro-4-nitrophenyl)naphthalene **(26a)**

Brown/orange solid, 74.4%, 226.0 mg. ^1^H NMR (400 MHz, Chloroform-*d*) δ 8.20 (t, *J* = 8.1 Hz, 1H), 7.95 (d, *J* = 8.4 Hz, 2H), 7.79 (d, *J* = 8.2 Hz, 1H), 7.59–7.48 (m, 3H), 7.47–7.40 (m, 3H). ^13^C NMR (100 MHz, Chloroform-*d*) δ 155.39 (d, *J* = 265.5 Hz), 149.21 (d, *J* = 8.5 Hz), 136.51 (d, *J* = 1.4 Hz), 133.78, 130.59, 129.46, 128.69, 127.07 (d, *J* = 9.4 Hz), 126.40, 126.19 (d, *J* = 3.8 Hz), 126.09 (d, *J* = 2.4 Hz), 125.29, 124.79, 119.90, 119.69.

#### 5-(naphthalen-1-yl)-2-nitroaniline **(26b)**

Brown/yellow solid, 99%%. ^1^H NMR (400 MHz, Chloroform-*d*) δ 8.23 (d, *J* = 8.8 Hz, 1H), 7.93–7.85 (m, 3H), 7.54–7.44 (m, 3H), 7.41 (d, *J* = 6.9 Hz, 1H), 6.92 (d, *J* = 1.7 Hz, 1H), 6.85 (dd, *J* = 8.8, 1.3 Hz, 1H), 6.15 (s, 2H). ^13^C NMR (100 MHz, DMSO-*d6*) δ 147.85, 146.58, 138.21, 133.77, 130.62, 129.99, 128.99, 128.91, 127.13, 126.86, 126.62, 126.02, 125.93, 125.41, 120.23, 117.99.

#### 3-fluoro-4’-methoxy-4-nitro-1,1‘-biphenyl **(27a)**

Pale orange solid, 82%, 231 mg. ^1^H NMR (400 MHz, Chloroform-*d*) δ 8.16–8.10 (m, 1H), 7.59–7.54 (m, 2H), 7.48–7.41 (m, 2H), 7.04–6.99 (m, 2H), 3.88 (s, 3H). ^13^C NMR (100 MHz, Chloroform-*d*) δ 160.89, 156.1 (d, *J* = 264.3 Hz), 148.82 (d, *J* = 8.7 Hz), 129.69 (d, *J* = 1.8 Hz), 128.95, 128.48, 126.66 (d, *J* = 2.4 Hz), 122.07 (d, *J* = 3.5 Hz), 115.64 (d, *J* = 21.7 Hz), 114.71, 55.44.

#### 4’-methoxy-4-nitro-[1,1’-biphenyl]-3-amine **(27b)**

Orange solid, 99%%. ^1^H NMR (400 MHz, Chloroform-*d*) δ 8.16 (d, *J* = 8.9 Hz, 1H), 7.53 (d, *J* = 8.8 Hz, 2H), 6.99 (d, *J* = Hz, 2H), 6.93 (d, *J* = 1.8 Hz, 1H), 6.91 (dd, *J* = 8.9, 1.8 Hz, 1H), 6.12 (s, 2H), 3.86 (s, 3H). ^13^C NMR (101 MHz, Chloroform-d) δ 160.38, 148.08, 144.92, 131.15, 128.50, 128.35, 126.82, 116.00, 115.60, 114.39, 55.39.

#### 2-(2-fluoro-5-nitrophenyl)thiophene **(28a)**

Yellow/green solid, 22%, 55.3 mg. ^1^H NMR (400 MHz, Chloroform-*d*) δ 8.22 (d, *J* = 2.8 Hz, 1H), 8.08 (dd, *J* = 9.1, 2.8 Hz, 1H), 7.36 (dd, *J* = 5.1, 1.0 Hz, 1H), 7.28 (dd, *J* = 3.6, 1.0 Hz, 1H), 7.07 (dd, *J* = 5.1, 3.6 Hz, 1H), 6.98 (d, *J* = 9.1 Hz, 1H). ^13^C NMR (100 MHz, Chloroform-*d*) δ 156.61, 141.66 (d, *J* = 208.4 Hz), 140.92, 128.50, 128.38 (d, *J* = 18.9 Hz), 128.31 (d, *J* = 12.9 Hz), 127.46, 126.92, 126.25 (d, *J* = 0.7 Hz), 124.07.

#### 2,4’-difluoro-5-nitro-1,1’-biphenyl **(29a)**

Yellow solid, 54%, 144 mg. ^1^H NMR (400 MHz, Chloroform-*d*) δ 8.09 (dd, *J* = 9.1, 2.7 Hz, 1H), 8.01 (d, *J* = 2.7 Hz, 1H), 7.49–7.41 (m, 2H), 7.16–7.08 (m, 2H), 6.92 (d, *J* = 9.1 Hz, 1H). ^13^C NMR (100 MHz, Chloroform-*d*) δ 162.06 (d, *J* = 247.2 Hz), 156.08, 142.68, 136.47 (d, *J* = 3.5 Hz), 129.84 (d, *J* = 7.9 Hz), 128.43 (d, *J* = 11.7 Hz), 128.08, 124.21, 115.88 (d, *J* = 1.7 Hz), 115.66.

#### 1-(2-fluoro-5-nitrophenyl)naphthalene **(30a)**

Yellow solid, 51%, 153.3 mg. ^1^H NMR (400 MHz, Chloroform-d) δ 8.18 (dd, *J* = 9.2, 2.8 Hz, 1H), 8.09 (d, *J* = 2.8 Hz, 1H), 7.91–7.84 (m, 2H), 7.59 (d, *J* = 8.3 Hz, 1H), 7.54–7.38 (m, 4H), 6.92 (d, *J* = 9.2 Hz, 1H). ^13^C NMR (101 MHz, Chloroform-d) δ 156.00, 138.71, 138.40, 133.68, 131.13, 130.53, 129.57, 128.98, 128.40, 128.33 (d, *J* = 28.4 Hz), 127.12, 126.63 (d, *J* = 244.0 Hz) 126.45, 126.09, 125.56 (d, *J* = 6.1 Hz), 124.63.

#### 4-nitrophenol **(31b)**

White solid, 84%%. ^1^H NMR (400 MHz, Chloroform-*d*) δ 8.19 (d, *J* = 9.1 Hz, 2H), 6.93 (d, *J* = 9.1 Hz, 2H), 5.84 (s, 1H). ^13^C NMR (100 MHz, Chloroform-*d*) δ 161.65, 141.43, 126.31, 115.73.

#### 4-bromo-2-nitrophenol **(32b)**

Brown solid, 86%. ^1^H NMR (400 MHz, Chloroform-*d*) δ 10.49 (s, 1H), 8.26 (d, *J* = 2.4 Hz, 1H), 7.67 (dd, *J* = 8.9, 2.4 Hz, 1H), 7.08 (d, *J* = 8.9 Hz, 1H). ^13^C NMR (100 MHz, Chloroform-*d*) δ 154.10, 140.33, 127.31, 121.72, 111.69.

#### 4-nitro-2-(trifluoromethyl)phenol **(33b)**

White solid, 70δ. ^1^H NMR (400 MHz, DMSO-*d6*) δ 9.50 (b, 1H), 8.57 (s, 1H), 8.35–8.31 (m, 18H), 7.14 (d, *J* = 9.2 Hz, 11H). ^13^C NMR (100 MHz, DMSO-*d6*) δ 170.28, 166.60, 139.43, 130.45, 126.94, 118.93, 114.63.

#### 2-nitro-4-(trifluoromethyl)phenol **(35b)**

Brown solid, 35%%. ^1^H NMR (400 MHz, DMSO-*d6*) δ 12.55 (b, 1H), 8.34 (d, *J* = 2.1 Hz, 1H), 8.02 (dd, *J* = 8.7, 2.1 Hz, 1H), 7.18 (d, *J* = 8.7 Hz, 1H). ^13^C NMR (100 MHz, DMSO-*d6*) δ 166.09, 156.39, 137.14, 135.81, 127.30, 121.72, 119.84.

#### benzene-1,4-diamine **(36b)**

Dark solid, 99%%. ^1^H NMR (400 MHz, CDCl_3_) δ 6.60 (s, 1H), 3.22 (s, 1H). ^13^C NMR (100 MHz, CDCl_3_) δ 138.60, 116.74.

#### benzene-1,2-diamine **(37b)**

Dark solid, 99%%. ^1^H NMR (400 MHz, CDCl_3_) δ 6.78–6.71 (m, 4H), 3.33 (s, 4H).

#### 2-fluorobenzene-1,4-diamine **(38b)**

Dark solid, 76%%. ^1^H NMR (400 MHz, CDCl_3_) δ 6.68–6.63 (m, 1H), 6.47–6.43 (m, 1H), 6.37–6.35 (m, 1H), 3.38 (s, 4H). ^13^C NMR (100 MHz, CDCl_3_) δ 126.02 (d, *J* = 13.3 Hz), 118.49 (d, *J* = 4.6 Hz), 116.74, 111.49 (d, *J* = 3.2 Hz), 103.59 (d, *J* = 22.2 Hz).

#### 2-(trifluoromethyl)benzene-1,4-diamine **(39b)**

Dark solid, 99%%. ^1^H NMR (400 MHz, CDCl_3_) δ 6.84 (d, *J* = 2.6 Hz, 1H), 6.78–6.69 (m, 1H), 6.65 (d, *J* = 8.5 Hz, 1H), 3.65 (s, 4H).

#### 2-methoxybenzene-1,4-diamine **(40b)**

Dark solid, 89%%. ^1^H NMR (400 MHz, CDCl_3_) δ 6.59 (d, *J* = 8.1 Hz, 1H), 6.29 (d, *J* = 2.4 Hz, 1H), 6.22 (dd, *J* = 8.1, 2.4 Hz, 1H), 3.83 (s, 3H), 3.21 (s, 4H). ^13^C NMR (100 MHz, CDCl_3_) δ 148.50, 139.01, 128.20, 116.37, 107.68, 100.12, 55.42.

## Figures and Tables

**Table 1.** Optimization studies of **1a**.^a^ 
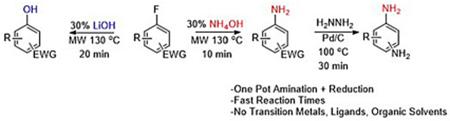

Entry	Temp (°C)	Time (min.)	Conv. (%)^b^
1	70	10	11
2	90	10	50
3	110	10	100
4	130	10	100
5	130	5	100

a Conditions: 100 mg **1a** added to 2 mL solvent. Heated in a sealed vessel in microwave.

b Determined by ^1^HNMR.

**Table 2.** Synthesis of amines from aryl halides^a^.

Entry	Subst.	Prod.	Conv.^b^ (%)	Yield (%)	Entry	Subst.	Prod.	Conv.^b^ (%)	Yield (%)
1	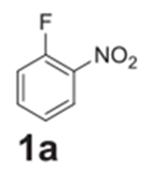	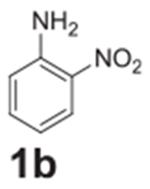	100	93	10	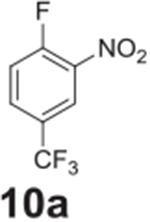	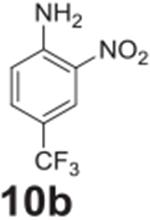	100	99
2	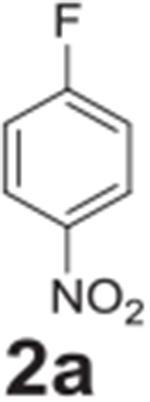	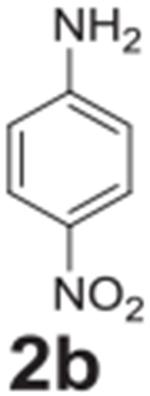	100	94	11	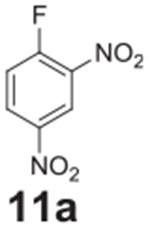	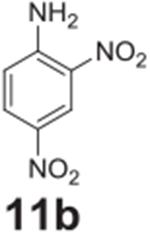	100	96
				N/A					
3	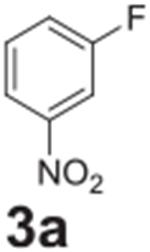	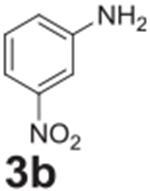	0		12^c, d^	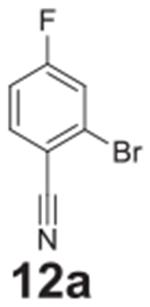	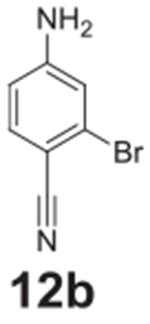	80	78
4	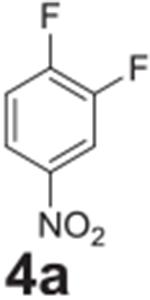	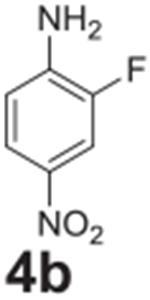	100	98	13	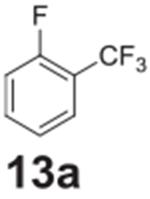	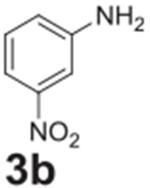	0	N/A
5	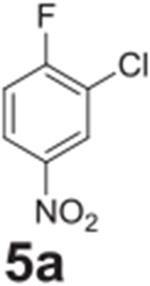	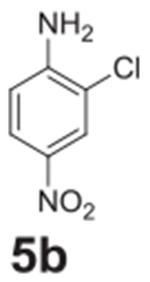	100	99	14	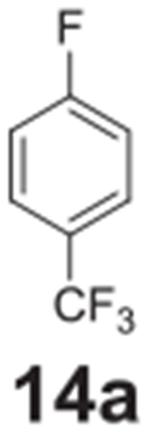	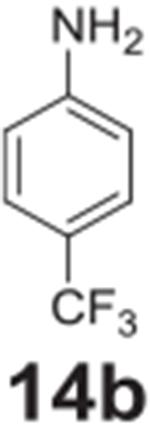	0	N/A
6	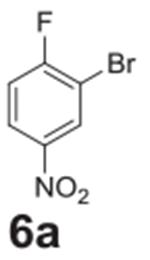	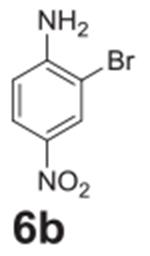	100	86	15^c, d^	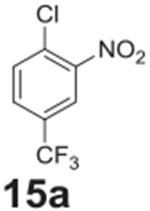	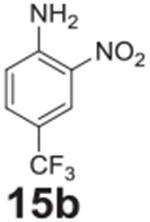	100	90
7	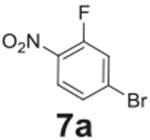	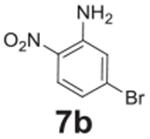	100	97	16^c, d^	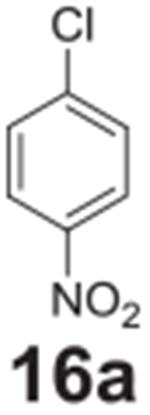	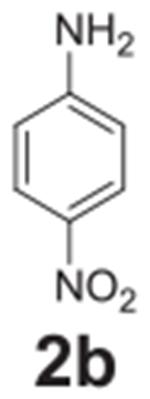	20	15
8	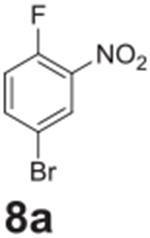	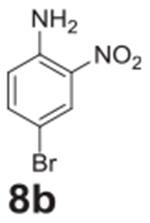	100	96	17	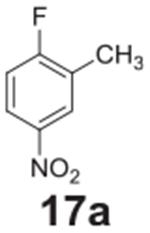	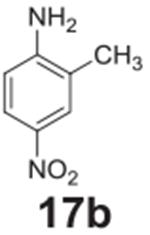	100	99
9	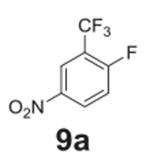	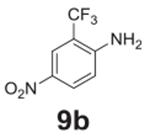	100	84	18	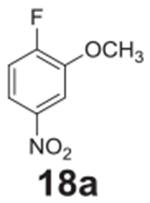	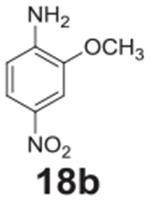	100	84

a Conditions: Unless otherwise stated, 100 mg starting material added to 2 mL of 30% NH_4_OH. Heated in microwave at 130°C for 10 min.

b Determined by ^1^HNMR.

c Heated at 140°C.

d Heated for 20 min.

**Table 3.** Synthesis of anilines from heterocycle substituted aryl halides^a^.

Entry	Subst.	Prod.	Conv.^b^ (%)	Yield (%)	Entry	Subst.	Prod.	Conv.^b^ (%)	Yield (%)
1	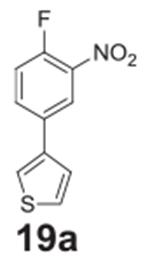	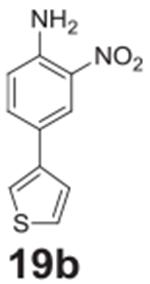	100	99	7^c^	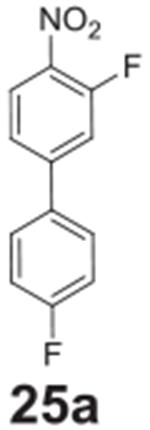	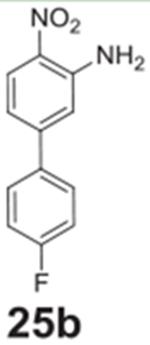	100	99
2	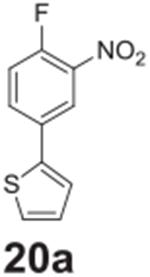	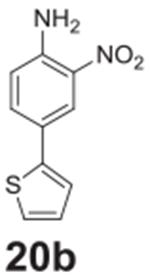	100	99	8^c^	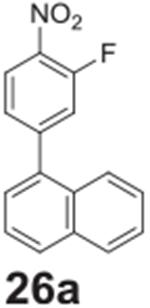	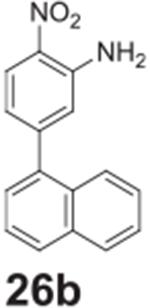	100	99
3	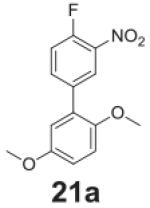	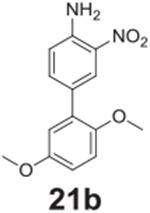	100	99	9^c^	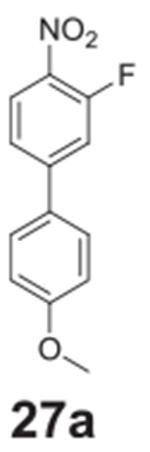	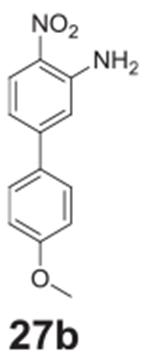	100	99
4	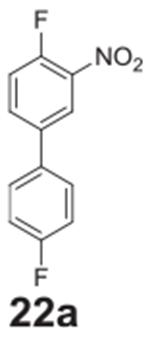	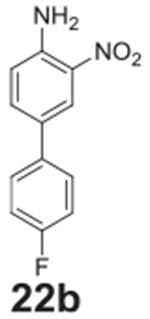	100	99	10^c, d^	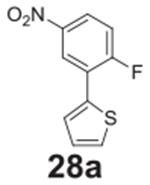	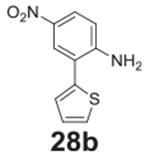	0	N/A
5	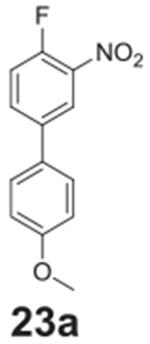	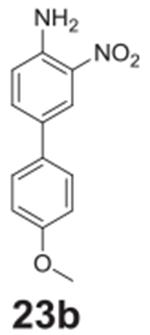	100	99	11^c, d^	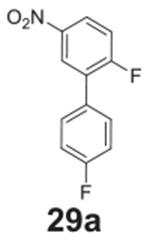	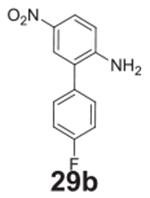	0	N/A
6	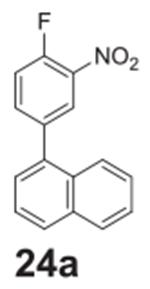	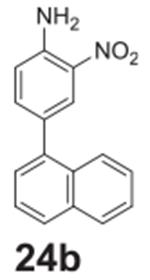	100	99	12^c, d^	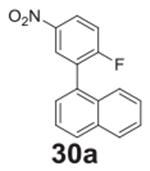	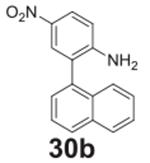	0	N/A

a Conditions: Unless otherwise stated, 50 mg starting material added 1 mL of 30% NH_4_OH. Heated in microwave at 130°C for 10 min.

b Determined by ^1^HNMR.

c Heated for 20 min.

d Heated at 140°C.

**Table 4.** Synthesis of phenols from aryl halides^a^.

Entry	Substrate	Product	Conv.^b^ (%)	Yield (%)
1	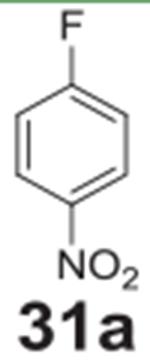	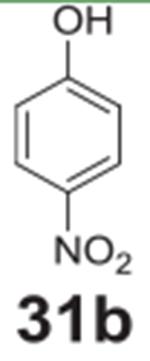	100	84
2	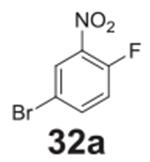	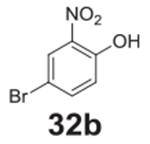	100	86
3	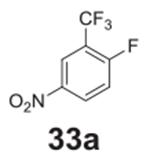	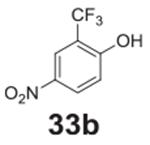	100	70
4^c, d^	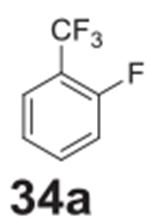	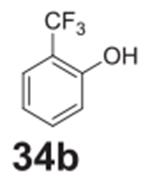	0	N/A
5^c, d^	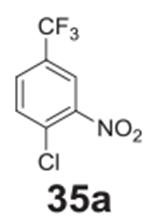	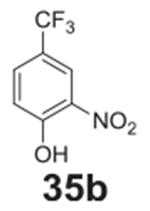	100	35

a Conditions: Unless otherwise stated, 100 mg starting material added 2 mL of 30% LiOH. Heated in microwave at 130°C for 20 min.

b Determined by ^1^HNMR.

c Heated at 140°C.

d Heated for 20 min.

**Table 5.** Synthesis of benzenediamines from aryl halides.

Entry	Substrate	Product	Conv.^a^ (%)	Yield (%)
1	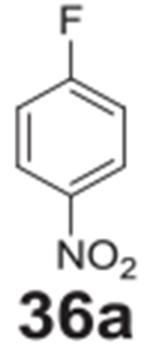	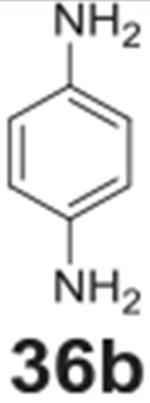	100	99
2	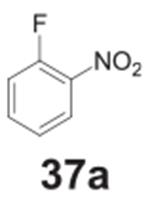	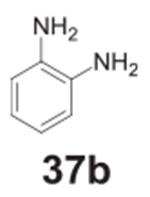	100	99
3	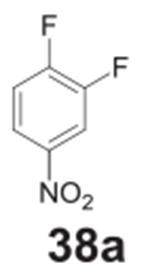	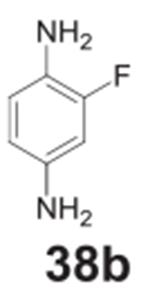	100	76
4	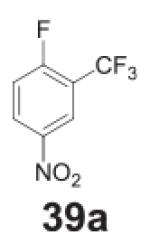	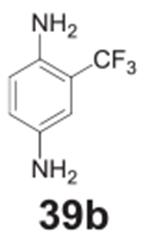	100	99
5	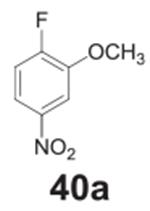	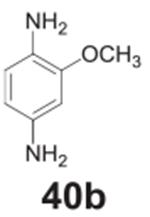	100	89

a Determined by ^1^HNMR.
